# Supplementary material for: The V2 Protein from the Geminivirus Tomato Yellow Leaf Curl Virus Largely Associates to the Endoplasmic Reticulum and Promotes the Accumulation of the Viral C4 Protein in a Silencing Suppression-Independent Manner
Source: Viruses. 2022 Dec 15;14(12):2804. doi: 10.3390/v14122804 (PMC9784378; doi:10.3390/v14122804)
Supplement: Supplementary file 1 [file viruses-14-02804-s001.zip › Wang et al., 2022 Supplementary Figures and Tables.pdf]

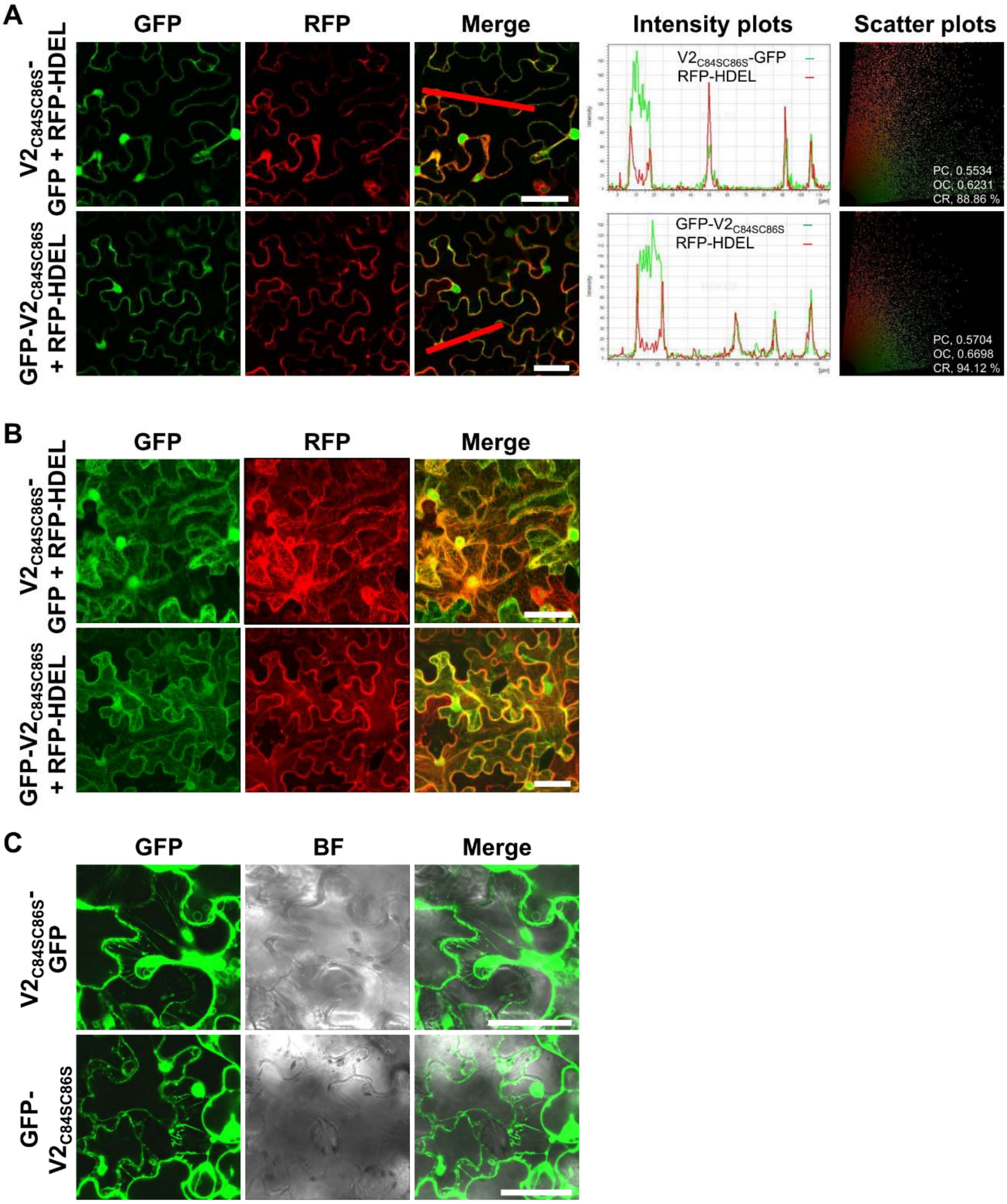

Supplementary Figure S1. Subcellular localization of V2$_{C84S/C86S}$ fused to GFP in *N. benthamiana* epidermal cells. *N. benthamiana* leaves were infiltrated with *A. tumefaciens* carrying constructs to express the ER marker protein RFP-HDEL and GFP-fused V2$_{C84S/C86S}$ (A and B) or infiltrated with *A. tumefaciens* carrying constructs to express GFP-fused V2$_{C84S/C86S}$ alone (C). Colocalization analysis (A) and maximum projection views of Z-stack images (B) of GFP fused V2$_{C84S/C86S}$ and RFP-HDEL, observed under the confocal microscope at 2 days post-infiltration (dpi). Colocalization between the GFP and RFP channels is analyzed with intensity plots (under the red line of the merge image) and scatter plots. (PC: Pearson's Correlation, OC: Overlap Coefficient, CR: Co-localization Rate.) (C) Plasmolysis of *N. benthamiana* leaves expressing GFP-fused V2$_{C84S/C86S}$ was observed under the confocal microscope 15 minutes after 1 M NaCl treatment. These experiments were repeated three times with similar results; representative images are shown. Scale bar: 50 μm.

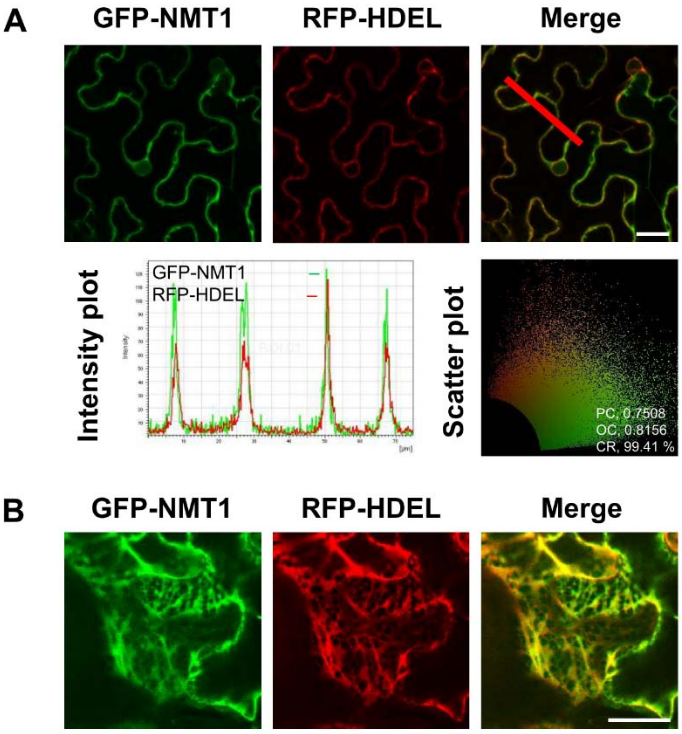

Supplementary Figure S2. Subcellular localization of NMT1 in *N. benthamiana* epidermal cells.
N. benthamiana leaves were infiltrated with *A. tumefaciens* carrying constructs to express the ER marker protein RFP-HDEL and GFP-NMT1 (A and B). Colocalization analysis (A) and close-up images (B) of GFP-NMT1 and RFP-HDEL were observed under the confocal microscope at 2 days post-infiltration (dpi). Colocalization between the GFP and RFP channels was analyzed with an intensity plot (under the red line of the merge image) and scatter plot. (PC: Pearson's Correlation, OC: Overlap Coefficient, CR: Co-localization Rate.) This experiment was repeated three times with similar results; representative images are shown. Scale bar: 25 μm.

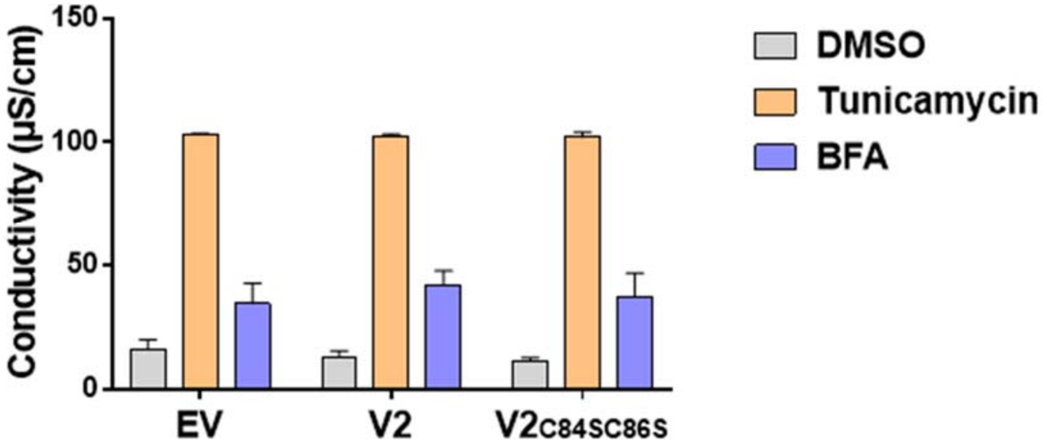

Supplementary Figure S3. Effect of tunicamycin or BFA treatment on cell death in *N. benthamiana* leaves expressing V2, $V2_{C84S/C86S}$, or transformed with an empty vector.

N. benthamiana leaves were infiltrated with *A. tumefaciens* carrying constructs to express V2, $V2_{C84S/C86S}$, or an empty vector as negative control. One day after *A. tumefaciens*-mediated infiltration, the same leaves were treated with the endoplasmic reticulum (ER) stress-inducing chemical (20 µg/mL TM or 30 µg/mL BFA) or 0.2% DMSO (as control). The extent of cell death was measured quantitatively by monitoring electrolyte leakage. Conductivity of leaf discs was measured after 24 hours. Values are the mean of four biological repeats; error bars indicate SEM.

Supplementary Table S1: Primers used in this work.

(1) pENTR/TOPO cloning		
Plasmid name	Vector name (Source)	Primer sequence
pENTR/TOPO-NMT1 (with stop codon)	pENTR/D-TOPO (Thermo Scientific)	F: CACCATGGCAGATAACAATTCACC<br>R: TTATAAGAGAACAAGCCCGAGTTCCGC
(2) Mutagenesis		
pENTR/TOPO-V2C84S/C86S (without stop codon)	pENTR/TOPO-V2 (without stop codon)	F:GCCCATACAGCAGCCGAGCTGCAG-TCCCCATTGTCCAAGG<br>R:CCTTGGACAATGGGGACTG-CAGCTCGGCTGCTGTATGGGC
pENTR/TOPO-V2C84S/C86S (with stop codon)	pENTR/TOPO-V2 (with stop codon)	
(3) Primers for RT-qPCR.		
qRep-1-F	TGAGAACGTCGTGTCTTCCG	
qRep-1-R	TGACGTTGTACCACGCATCA	
qC4-1-F	CAACGGTTCTTCGACCTGGT	
qC4-1-R	GGGCCTCGGATTTATTGCCT	
qGFP-F	TATATCATGGCCGACAAGCA	
qGFP-R	GAACTCCAGCAGGACCATGT	

Supplementary Table S2. List of plasmids generated in this study.

Plasmid name	Entry vector name (Source)	Binary vector (Source)
RFP-GFP	TOPO-GFP (with stop codon) [26]	pGWB555 [46]
V2C84SC86S	TOPO-V2C84SC86S (with stop codon) (This study)	pGWB2 [47]
V2C84SC86S-GFP	TOPO-V2C84SC86S (without stop codon) (This study)	pGWB505 [46]
GFP-V2C84SC86S	TOPO-V2C84SC86S (with stop codon) (This study)	pGWB506 [46]
GFP-NMT1	TOPO-NMT1 (with stop codon) (This study)	pGWB555 [46]
